# Brg1 and RUNX1 synergy in regulating TRPM4 channel in mouse cardiomyocytes

**DOI:** 10.3389/fphar.2024.1494205

**Published:** 2024-12-12

**Authors:** Tao Ban, Xianhui Dong, Ziyue Ma, Jing Jin, Jing Li, Yunfeng Cui, Yuyang Fu, Yongzhen Wang, Yadong Xue, Tingting Tong, Kai Zhang, Yuxuan Han, Meimei Shen, Yu Zhao, Ling Zhao, Lingzhao Xiong, Hongzhao Lv, Yang Liu, Rong Huo

**Affiliations:** ^1^ Harbin Medical University and Department of Pharmacology (State Key Laboratory of Frigid Zone Cardiovascular Diseases, Ministry of Science and Technology; State Key Labratoray-Province Key Laboratories of Biomedicine-Pharmaceutics of China, Key Laboratory of Cardiovascular Research, Ministry of Education) at College of Pharmacy, Harbin, China; ^2^ Heilongjiang Academy of Medical Sciences, Harbin, China; ^3^ Department of General Surgery, The Fourth Affiliated Hospital of Harbin Medical University, Harbin, China

**Keywords:** TRPM4, BRG1, Runx1, hypoxia, cardiomyocyte

## Abstract

**Background:**

Transient Receptor Potential Melastatin 4 (TRPM4), a non-selective cation channel, plays a critical role in cardiac conduction abnormalities. Brg1, an ATP-dependent chromatin remodeler, is essential for regulating gene expression in both heart development and disease. Our previous studies demonstrated Brg1 impacted on cardiac sodium/potassium channels and electrophysiological stability, its influence on TRPM4 expression and function remained unexplored.

**Methods:**

We investigated the role of Brg1 in regulating TRPM4 expression and function through overexpression and knockdown experiments in mouse cardiomyocytes and TRPM4-overexpressing HEK293 cells by western blot, qPCR, immunofluorescence staining and patch clamp techniques. Cardiomyocytes were exposed to hypoxia for 12 h to mimic cardiac stress, and Brg1 inhibition was performed to assess its impact on TRPM4 under hypoxia. Bioinformatic analyses (STRING and JASPAR databases), Co-immunoprecipitation (Co-IP), dual luciferase reporter assays, and Chromatin Immunoprecipitation (ChIP) were employed to study the interaction between Brg1, RUNX1, and TRPM4 transcription regulation.

**Results:**

Brg1 positively regulated TRPM4 expression in mouse cardiomyocytes and modulated TRPM4 current in TRPM4-overexpressing HEK293 cells. Brg1 inhibition markedly diminishes TRPM4’s hyperexpression in cardiomyocytes exposed to hypoxia. Integrative analyses utilizing STRNG databases and Protein Data Bank unveiled a putative interaction between Brg1 and the transcription factor RUNX1, and we substantiated the interaction between Brg1 and RUNX1. Several binding sites of RUNX1 with the TRPM4 promoter region were predicted by the JASPAR database, and empirical validation substantiated Brg1 modulated TRPM4 promoter activity via RUNX1 engagement. ChIP confirmed that Brg1 interacted with RUNX1 forming a transcriptional complex that located in TRPM4 promoter.

**Conclusion:**

Our study demonstrated that Brg1 and RUNX1 formed a transcriptional complex that modulated TRPM4 expression and function, especially under hypoxic conditions. These findings provided new insights into TRPM4 regulation and highlighted its potential as a therapeutic target for cardiac hypoxia-related disorders.

## 1 Introduction

The Transient Receptor Potential Melastatin-like subfamily member 4 (TRPM4) stands out as a voltage-dependent, non-selective monovalent cation channel, which is activated by Ca^2+^ ions and is encoded by the gene for TRPM4 (Wang et al., 2018; Montell, 2005; Yu et al., 2019). Characterized by the presence of six transmembrane domains, similar to other channels within the TRP family (Guinamard et al., 2010; Vennekens and Nilius, 2007), the three-dimensional structure of TRPM4 has been elucidated, confirming its formation as a tetrameric protein complex (Guinamard et al., 2010; Vennekens and Nilius, 2007; Winkler et al., 2017; Autzen et al., 2018; Guo et al., 2017). In general, TRPM4 facilitates equal permeability to Na^+^ and K^+^ ions while excluding Ca^2+^ ions (Guinamard et al., 2004; Kecskés et al., 2015). The expression of TRPM4 across diverse tissues, such as the brain, heart, kidney, colon, and intestine, signifies its crucial involvement in a wide range of physiological and pathological processes (Winkler et al., 2017; Earley et al., 2004). TRPM4 regulated many physiological processes include insulin secretion, constriction of cerebral arteries, modulation of immune and respiratory responses, tumorigenesis, and the central nervous system (Kaneko and Szallasi, 2014; Kruse and Pongs, 2014). Moreover, TRPM4 also plays an important role in the development of cardiomyocytes and multiple cardiovascular diseases, including endothelial injury, myocardial ischemia, myocardial hypertrophy, arrhythmias, heart failure, etc*.* (Wang et al., 2018; Guinamard et al., 2015; Becerra et al., 2011). In cardiac electrophysiology, TRPM4 channels influence membrane potential to alter excitability through participation in membrane voltage regulation of intracellular Ca^2+^ (Guinamard et al., 2015; Abriel et al., 2012; Guinamard et al., 2014; Mathar et al., 2014), which is closely related to arrhythmias. Several lines of evidence suggest that TRPM4 is closely related to arrhythmias. On the one hand, mutations in the TRPM4 gene cause a variety of arrhythmic phenotypes including cardiac conduction block (Liu et al., 2010), long QT syndrome (Hof et al., 2017) and Brugada syndrome (Liu et al., 2013). On the other hand, TRPM4 expression is upregulated in a variety of cardiovascular diseases, mainly in atrial cardiomyocytes. This is associated with prolongation of the QT interval in the electrocardiogram (Guinamard et al., 2006), early after depolarization (EAD)-like oscillations during the repolarization phase of the action potential (AP) (Simard et al., 2012), and disruption of connexin-43 (Cx43) in the atria (Simard et al., 2021), which may act as pro-arrhythmic substrates for early and delayed depolarization.

The Brahma-related gene 1 (Brg1), functioning as an ATPase, is a crucial subunit of the SWI/SNF chromatin remodeling complexes. It is encoded by the *SMARCA4* gene (Ge et al., 2017; Matsumoto et al., 2016). These complexes harness the energy from ATP hydrolysis to bring about ATP-dependent changes in the structure and positioning of nucleosomes, significantly contributing to the complex process of gene transcription, and is involved in many processes, such as the formation of the preinitiation complex, along with the initiation and elongation of transcription (Barker et al., 2001; Debril et al., 2004; Soutoglou and Talianidis, 2002; Witwicka et al., 2019). Brg1 is essential in the development of critical organs, including the central nervous system, thymus, and heart, underlining its importance for mammalian growth and development (He et al., 2010). Furthermore, Brg1 meticulously regulates heart growth, differentiation, and gene expression in both normal and diseased states. Recent research has highlighted Brg1 as a promising therapeutic target for addressing pathological conditions such as myocardial hypertrophy and heart failure (Bevilacqua et al., 2014; Kidder et al., 2009; Ma et al., 2023; Li et al., 2024).

The TRPM4 inhibitor, 9-phenanthrene, has been demonstrated to effectively mitigate arrhythmias induced by ventricular hypoxia and reoxygenation in mice. Additionally, it can protect isolated rat hearts from ischemia-reperfusion injury (Simard et al., 2012; Pironet et al., 2019). Furthermore, previous research, including our own, has documented an increase in the expression of the Brg1 after myocardial infarction, highlighting its elevated expression in myocardial cells under cardiac stress (Bevilacqua et al., 2014; Hang et al., 2010). Additionally, in our previous study, we found that Brg1 affected multiple ion channels transcription level, such as Na_v_1.5 and K_v_4.3, that might be a potential mechanism to induce post-myocardial infarction arrhythmia (Li et al., 2024). These diseases often arise or worsen due to myocardial infarction and hypoxia-reoxygenation injury, among other stress conditions, making the exploration of the interaction between Brg1 and TRPM4 of paramount importance. Up to this moment, the question of whether Brg1 affects the TRPM4 channels has not been definitively answered. This study sets out to illuminate the regulatory influence of Brg1 on TRPM4 and the underlying mechanisms.

## 2 Results

### 2.1 Brg1 overexpression enhanced TRPM4 expression

**FIGURE 1:**
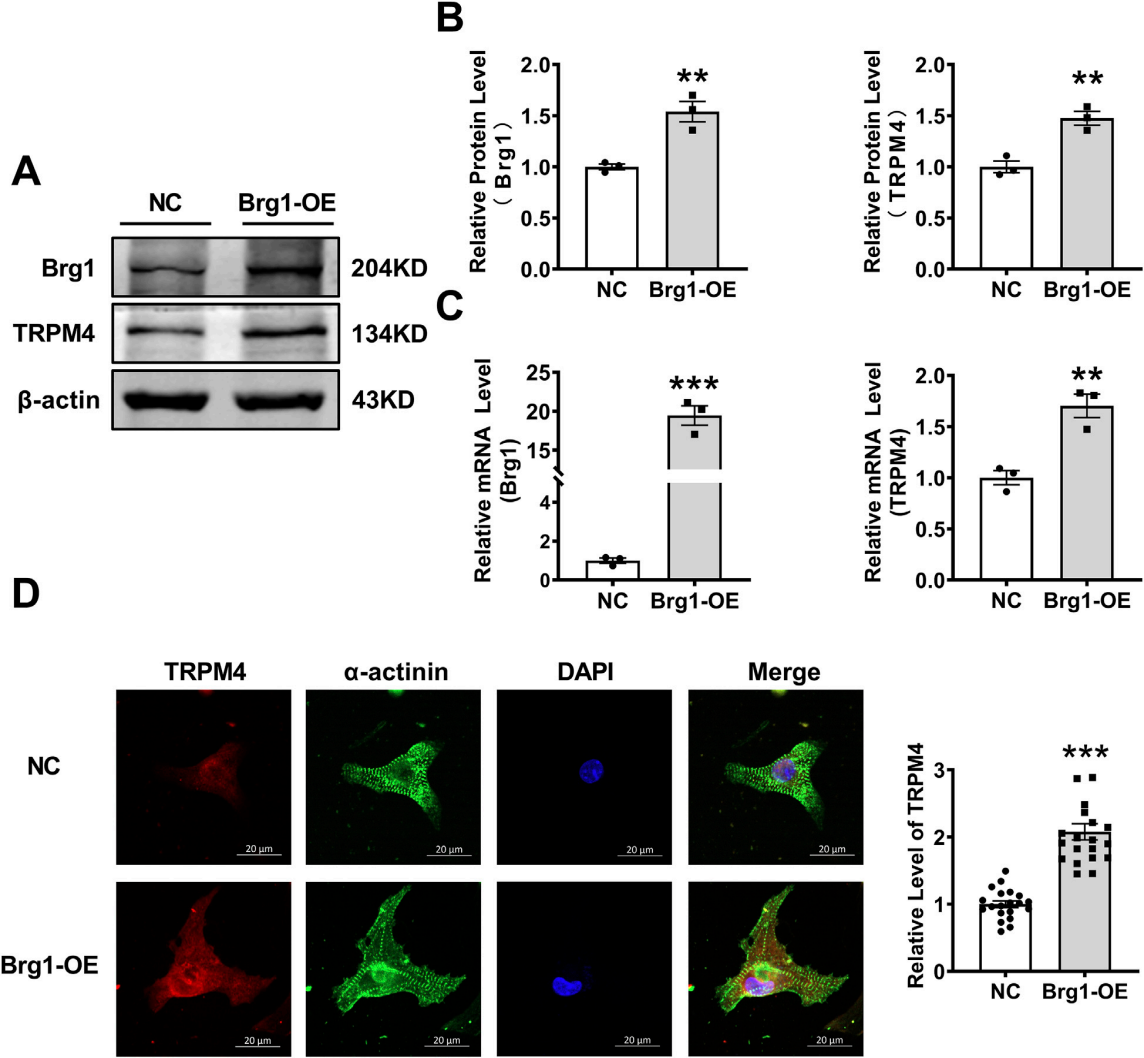
Brg1 overexpression increased the protein and mRNA levels of TRPM4 in neonatal mouse cardiomyocytes. **(A, B)** Brg1 overexpression increased TRPM4 protein level in neonatal mouse cardiomyocytes. Representative bands of Brg1 and TRPM4 protein were shown in **(A)** and the analyzed data was shown in **(B)**. n = 3 in each group. **(C)** Brg1 overexpression increased TRPM4 mRNA level in neonatal mouse cardiomyocytes. n = 3 in each group. **(D)** Immunofluorescent staining of TRPM4 and Brg1 were assessed after Brg1 overexpression in neonatal mouse cardiomyocytes. Scale bar indicates 20μm, n = 20 in each group. Statistical analysis was performed with two-tailed Student’s t-test. ^***^ *p* < 0.001 and ^**^ *p* < 0.01 vs. NC group.

In order to explore the potential relevance of Brg1 on TRPM4 channels. We constructed Brg1 overexpression (Brg1-OE) plasmid then transfected to neonatal mouse cardiomyocytes, thereby creating a model of Brg1 overexpression. After transfection of 48 h, we found that Brg1 mRNA and protein expression enhanced significantly. Meanwhile we observed a notable rise in the levels of TRPM4 mRNA and protein compared to the control group (Figures 1A–C). In addition, we further examined the effect of Brg1-OE on the TRPM4 fluorescence expression levels in the myocardial cells. As shown in Figure 1D, TRPM4 was located in both the membrane and the cytoplasm of cardiomyocytes, and Brg1 overexpression led to a significant increase in the fluorescence expression of TRPM4. These results indicated the positive regulatory effect of Brg1 on the expression of TRPM4 in cardiomyocytes.

### 2.2 Inhibition of Brg1 decreased TRPM4 expression

**FIGURE 2:**
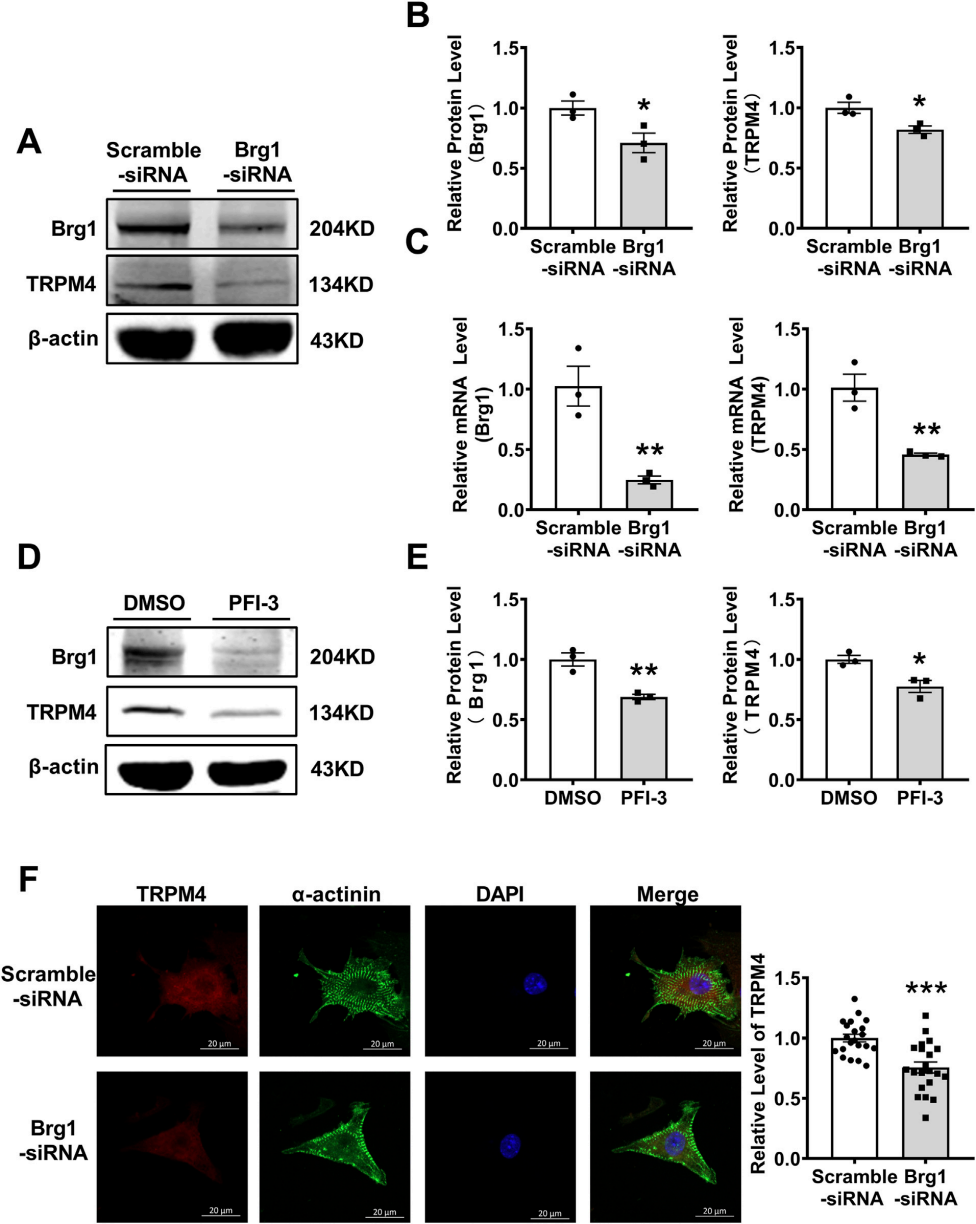
Brg1 knockdown decreased the protein and mRNA levels of TRPM4 in neonatal mouse cardiomyocytes. **(A, B)** Brg1 knockdown decreased TRPM4 protein level in neonatal mouse cardiomyocytes. Representative bands of Brg1 and TRPM4 protein were shown in **(A)** and the analyzed data was shown in **(B)**. ^*^ *p* < 0.05 vs. Scramble-siRNA group. n = 3 in each group. **(C)** Brg1 knockdown decreased TRPM4 mRNA level in neonatal mouse cardiomyocytes. ^**^ *p* < 0.01 vs. Scramble-siRNA group. n = 3 in each group. **(D)** Brg1 inhibition by PFI-3 also reduced TRPM4 protein level. Representative bands of Brg1 and TRPM4 protein were shown in **(D)** and the analyzed data was shown in **(E)**. ^**^ *p* < 0.01 and ^*^ *p* < 0.05 vs. DMSO group. n = 3 in each group. **(F)** Immunofluorescent staining of TRPM4 and Brg1 were assayed after Brg1 knockdown in neonatal mouse cardiomyocytes. Scale bar indicates 20 μm, n = 21 in each group. Statistical analysis was performed with two-tailed Student’s t-test.

Next, we want to further prove Brg1 and TRPM4 have regulatory effects by inhibition of Brg1. For this purpose, we silenced Brg1 in cardiomyocytes with a specific small interfering RNA (siRNA). The results showed Brg1-siRNA exhibited a notable decrement in both mRNA and protein levels of Brg1 compared with scramble-siRNA group, affirming the efficacy of the knockdown procedure (Figures 2A–C). We also found that TRPM4 mRNA and protein expression was decreased in Brg1-siRNA group compared to scramble-siRNA group. Meanwhile, we used PFI-3 further to measure the influence of Brg1 on the expression of TRPM4. PFI-3 is a small molecule inhibitor of Brg1, which not only affects Brg1 activity, but also inhibits Brg1 expression that have already been confirmed in our previous study (Li et al., 2024; Li et al., 2023) and relevant literature (Sharma et al., 2023). After PFI-3 treatment, the protein level of TRPM4 also reduced compared to DMSO-treated group (Figures 2D, E). Immunofluorescent staining demonstrated TRPM4 fluorescence levels declined when Brg1 knockdown (Figure 2F). Consistent with the changes in TRPM4’s mRNA and protein expressions after Brg1 inhibition.

### 2.3 Brg1 affects cell membrane potential in neonatal mouse cardiomyocytes by regulating TRPM4

**FIGURE 3:**
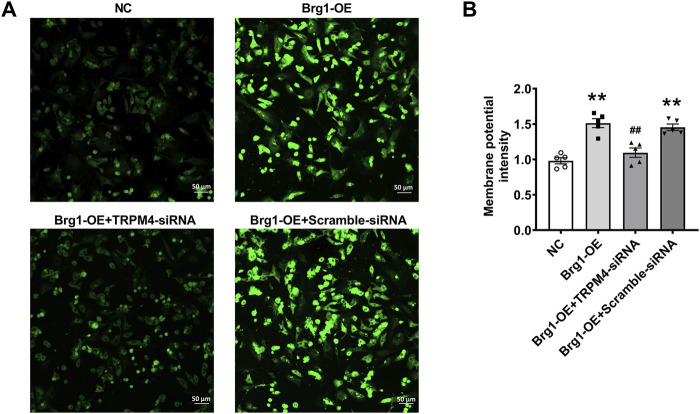
Brg1 affected cell membrane potential by regulating TRPM4 in neonatal mouse cardiomyocytes. Cell membrane potential in neonatal mouse cardiomyocytes was measured by using potentiometric fluorescence dye DiBAC4(3). Representative images of cell membrane potential were shown in **(A)**, and the analyzed data was shown in **(B)**. Scale bar indicates 50 μm n = 5 in each group from five batches of cells. Statistical analysis was performed with one-way ANOVA. **p < 0.01 vs. NC group; ^##^ *p* < 0.01 vs. Brg1-OE + Scramble-siRNA group.

In general, the continuous activation of TRPM4 channels in cardiomyocytes would cause depolarization and changes in membrane potential. In order to further verify that Brg1 regulate TRPM4 by exerting an influence on membrane potential, we utilized the DiBAC4 (Yu et al., 2019) fluorescent probe for the detection of cell membrane potential. This probe is well-known for its sensitivity in gauging the membrane potential of various biological systems. Increases of fluorescence intensity within the cytoplasm indicated of depolarization of the potential. Specifically, we observed Brg1 overexpression significantly increased the membrane potential in cardiomyocytes (Figure 3), which was reversed by TRPM4 siRNA treatment. These observations collectively suggested that Brg1 acted as a positive regulator of TRPM4 channels in neonatal mouse cardiomyocytes.

### 2.4 Brg1 overexpression enhanced TRPM4 current

**FIGURE 4:**
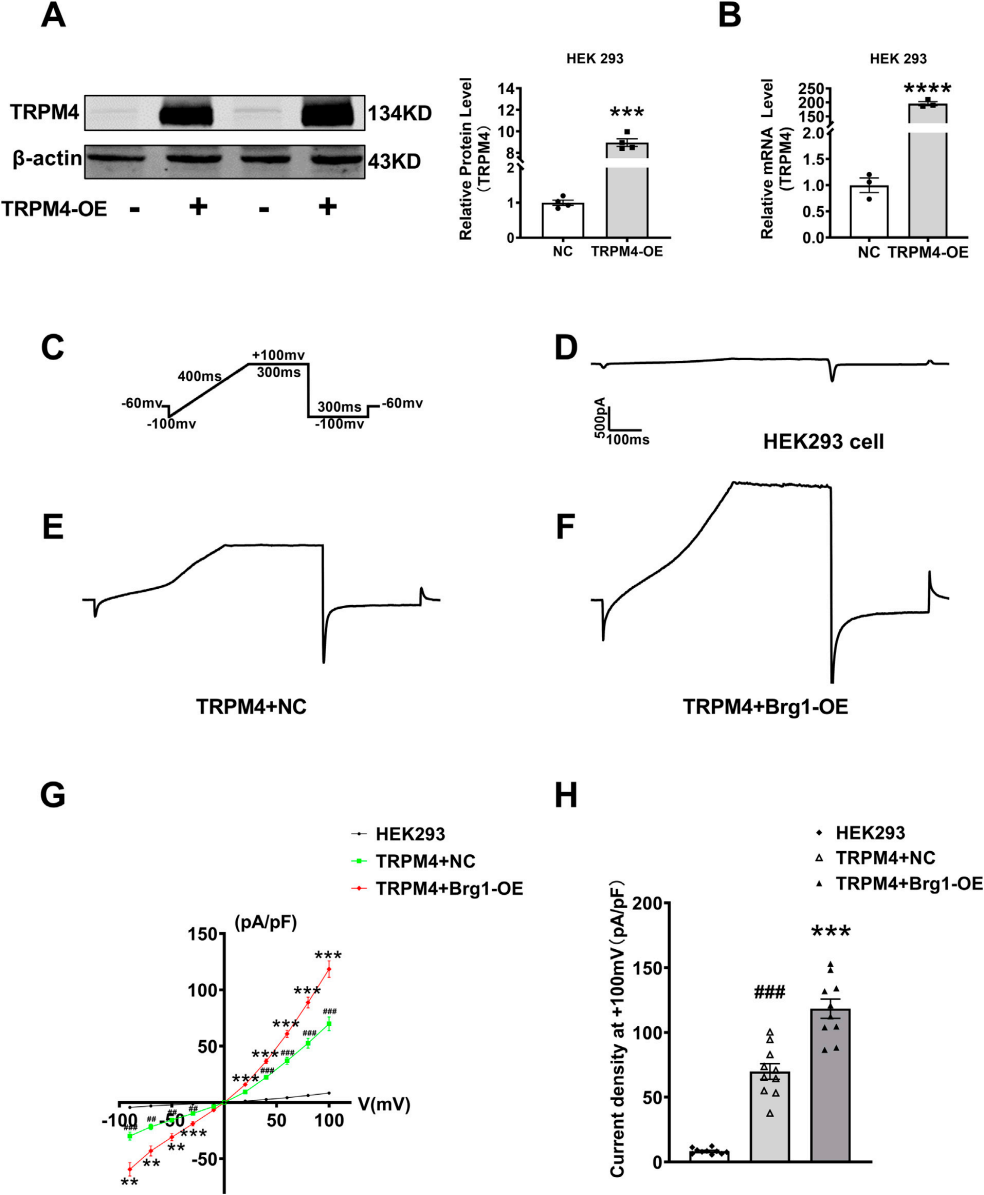
Brg1 overexpression increased TRPM4 current density in HEK293 cells. **(A–C)** We transfected plasmid contained full-length TRPM4 promoter region to HEK293 cells to construct TRPM4-overexpressed model for recording TRPM4 current. **(A)** Representative bands of TRPM4 protein in TRPM4-overexpressed model were on the left panel and the analyzed data was shown on the right panel. ^***^ *p* < 0.001 vs. NC group by a two-tailed Student’s t-test. n = 4 in each group. **(B)** TRPM4 mRNA level in TRPM4-overexpressed HEK293 cells. ^****^ *p* < 0.001 vs. NC group by a two-tailed Student’s t-test. n = 3 in each group. **(C)** Individual current recorded using a ramp protocol depicted in the inset. **(D–F)** Typical current traces of TRPM4 from HEK293 cells group, TRPM4+NC group and TRPM4+Brg1-OE group. **(G)** Current-voltage (*I-V*) relationship curves of TRPM4 from HEK293 cells group, TRPM4+NC group and TRPM4+Brg1-OE group. Statistical analysis was performed with one-way ANOVA. ^###^ *p* < 0.001 and ^##^ *p* < 0.01 vs. HEK293 cells group; ^***^ *p* < 0.001 and ^**^ *p* < 0.01 vs. TRPM4+NC group. n = 10 in each group. **(H)** Comparation of TRPM4 current density at +100mv from HEK293 cells group, TRPM4+NC group and TRPM4+Brg1-OE group. Statistical analysis was performed with one-way ANOVA. ^###^ *p* < 0.001 vs. HEK293 group; ^***^ *p* < 0.001 vs. TRPM4+NC group. n = 10 in each group.

Since TRPM4 is a non-selective monovalent cation channel, changes of TRPM4 expression would result in abnormal TRPM4 current in cardiovascular diseases (Liu et al., 2010; Hof et al., 2017; Liu et al., 2013). To this end, we used the whole-cell patch-clamp technique for recording of channel currents. To excluding the influence of other ion currents such as sodium or potassium current, we transfected plasmid contained full-length TRPM4 promoter region to construct a TRPM4-overexpressed model in HEK293 cells. After transfection 48 h, TRPM4 mRNA and protein levels enhanced strongly (Figures 4A, B). Then individual current recorded using a ramp protocol depicted in the inset (Figure 4C). Typical current traces of TRPM4 from HEK293 cells group, TRPM4+NC group and TRPM4+Brg1-OE group were shown in Figures 4D–F. These results showed a significant increment of TRPM4 current after TRPM4 plasmid transfected compared to HEK293 cell without transfection, while an additional increase was observed following Brg1 overexpression. In addition, *I-V* curves post-standardization (Figure 4G) and the current density of the cells at +100mv (Figure 4H) were analyzed in line with the above results. The findings clearly demonstrated an augmentation in TRPM4 current density in HEK293 cells subsequent to TRPM4 overexpression. Moreover, overexpression of Brg1 led to a further significant rise in TRPM4 current density. These results indicated that Brg1 overexpression not only boosted the expression but also the function of the TRPM4 channel.

### 2.5 Inhibition of Brg1 decreased TRPM4 current

**FIGURE 5:**
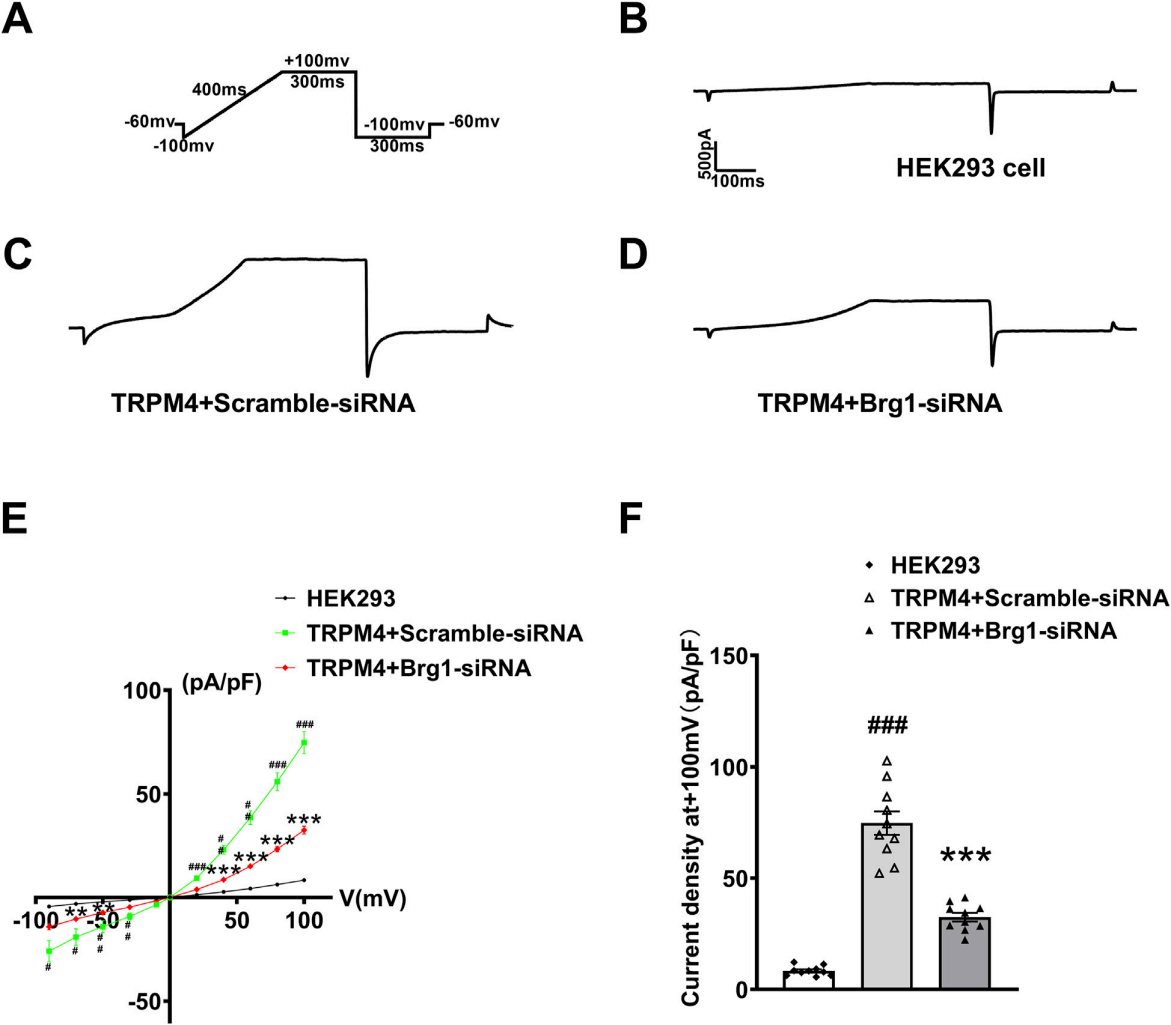
Brg1 knockdown decreased TRPM4 current density in HEK293 cells. **(A)** Individual current recorded using a ramp protocol depicted in the inset. **(B–D)** Typical current trace from HEK293 cell group, TRPM4+Scramble-siRNA group and TRPM4+Brg1-siRNA group. **(E)** Current-voltage (*I-V*) relationship curves of TRPM4 from HEK293 cells group, TRPM4+Scramble-siRNA group and TRPM4+Brg1-siRNA group. Statistical analysis was performed with one-way ANOVA. ^###^ *p* < 0.001, ^##^ *p* < 0.01 and ^#^ *p* < 0.05 vs. HEK293 group; ^***^ *p* < 0.001 and ^**^ *p* < 0.01 vs. TRPM4+Scramble-siRNA group. n = 10 in each group. **(F)** Comparation of TRPM4 current density at +100mv from HEK293 cells group, TRPM4+Scramble-siRNA group and TRPM4+Brg1-siRNA group. Statistical analysis was performed with one-way ANOVA. ^###^ *p* < 0.001 vs. HEK293 group; ^***^ *p* < 0.001 vs. TRPM4+Scramble-siRNA group. n = 10 in each group.

Next, we further silenced Brg1 in TRPM4-HEK293 cells to evaluate the effect of Brg1 on TRPM4 current. This cellular model served as a pivotal resource for recording TRPM4 currents under conditions of Brg1 knockdown. Illustrated in the figures, the experimental setup included a stimulation protocol (Figure 5A), and current traces were delineated for three distinct groups: HEK293 cells absent of TRPM4 transfection (Figure 5B), cells co-transfected with scramble-siRNA (Figure 5C), and cells co-transfected with Brg1-siRNA (Figure 5D). Notably, Comparison with the data observed in scramble-siRNA, the current of TRPM4 transfection group has significantly increased. In contrast, the knockdown of Brg1 led to a pronounced reduction in TRPM4 currents (Figures 5B–D).

Quantitative analysis yielded *I-V* curves post-normalization (Figure 5E) and the current density values for the cell groups at +100mv (Figure 5F). It was suggested that the increased current density was associated with TRPM4 overexpression in HEK293 cells. However, this elevated current density witnessed a substantial decline upon Brg1 knockdown. These observations robustly indicated that Brg1 inhibition not only diminished TRPM4 expression but also impaired its functional capacity. Hence, the regulation of Brg1 emerged as a crucial factor influencing both the expression and operational efficacy of the TRPM4 channel.

### 2.6 Brg1 inhibition regulated hypoxia-induced TRPM4 overexpression

**FIGURE 6:**
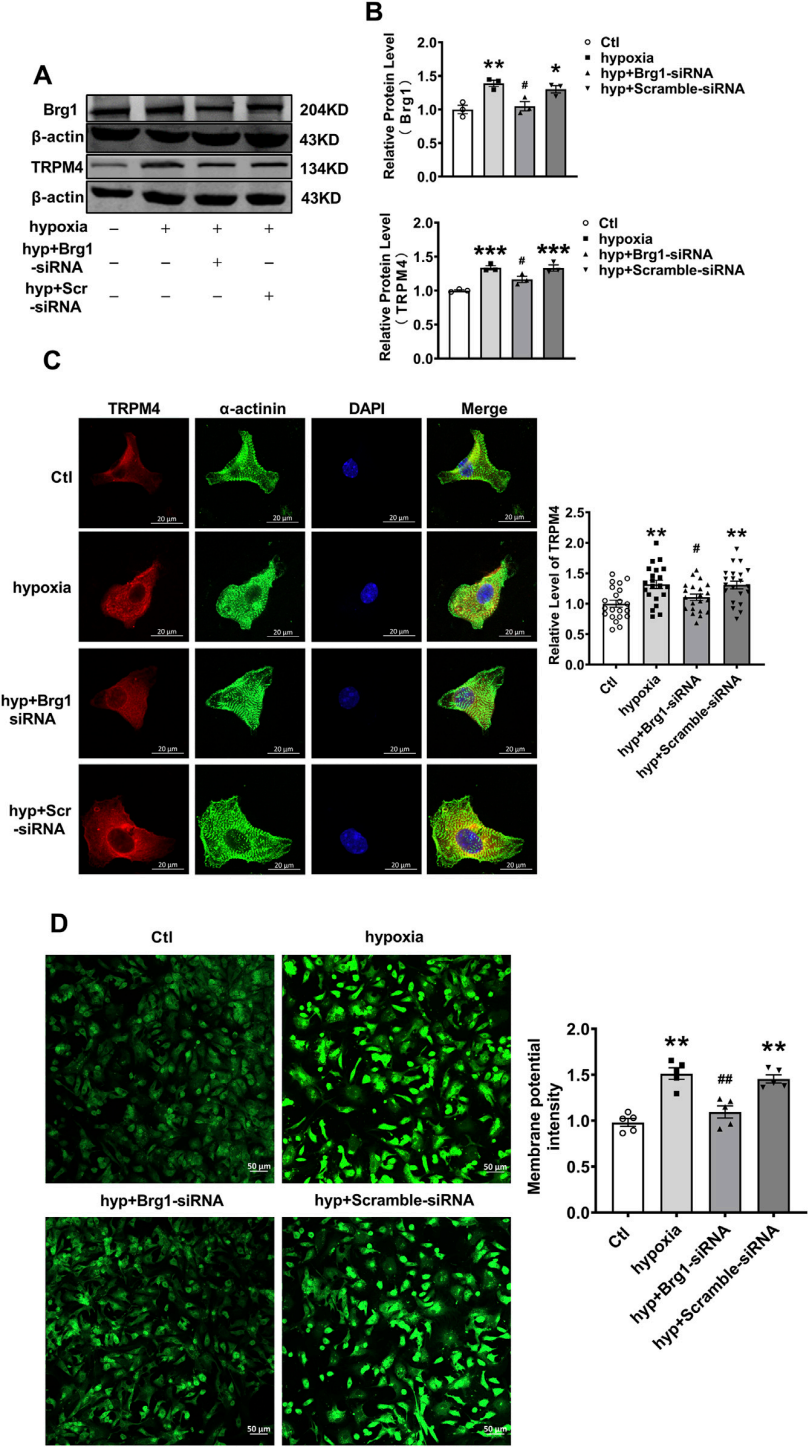
Brg1 knockdown restored partially hypoxia-induced aberrant high levels of TRPM4 expression in neonatal mouse cardiomyocytes. **(A)** The representative bands of Brg1 and TRPM4 protein from control group, hypoxia group, hyp + Brg1-siRNA group and hyp + Scramble-siRNA group. **(B)** The analyzed data of protein expression of Brg1 and TRPM4. Statistical analysis was performed with one-way ANOVA. ^*^ *p* < 0.05, ^**^ *p* < 0.01 and ^***^ *p* < 0.001 vs. Ctl group; ^#^ *p* < 0.05, vs. hyp + Scramble-siRNA group. n = 3 in each group. **(C)** Immunofluorescent staining of TRPM4 was assayed after hypoxia-treated for 12 h in neonatal mouse cardiomyocytes with or without Brg1 knockdown. Statistical analysis was performed with one-way ANOVA. ^**^ *p* < 0.01 vs. Ctl group; ^#^ *p* < 0.05 vs. hyp + Scramble-siRNA group. Scale bar indicates 20μm, n = 21 in each group from four batches of cells. **(D)** The representative image of the cell membrane potential in neonatal mouse cardiomyocytes on the left, and the analyzed data on the right. Statistical analysis was performed with one-way ANOVA. ^**^ *p* < 0.01 vs. Ctl group; ^##^ *p* < 0.01 vs. hyp + Scramble-siRNA group. Scale bar indicates 50μm, n = 5 in each group from five batches of cells.

The investigations thus far have primarily focused on TRPM4 in normoxic cardiomyocytes (Winkler et al., 2017; Earley et al., 2004). However, when cardiovascular diseases such as myocardial infarction as well as myocardial ischemia-reperfusion, cardiomyocytes were often subject to oxidative stress due to ischemia and hypoxia. Therefore, considering the prevalence of pathologic hypoxia and ischemia in various cardiac diseases, it was crucial to examine the effect of Brg1 on TRPM4 under hypoxic conditions. Most studies have used hypoxia as well as hydrogen peroxide-induced oxidative stress to mimic conditions such as ischemia and hypoxia *in vivo* (Li et al., 2024; Wang et al., 2019). To this end, after cardiomyocytes exposed to hypoxia for 12 h, both Brg1 and TRPM4 protein expression were significantly elevated. Intriguingly, Brg1 knockdown was found to effectively curb the hypoxia-triggered upsurge in TRPM4 expression (Figures 6A, B).

Further analysis through immunofluorescence staining showcased a pronounced increase in TRPM4 fluorescence in myocardial cells subjected to hypoxia. Remarkably, Brg1 suppression was observed to partially reverse the hypoxia-induced enhancement of TRPM4 fluorescence (Figure 6C). Additionally, we used the DiBAC4(3) fluorescent probe for the assessment of alterations in myocardial cell membrane potential under hypoxic stress. The findings indicated a notable escalation in cell membrane potential in response to hypoxia, the effect that was alleviated by Brg1 knockdown (Figure 6D).

### 2.7 Inhibition of Brg1 attenuated hypoxia-induced TRPM4 current

**FIGURE 7:**
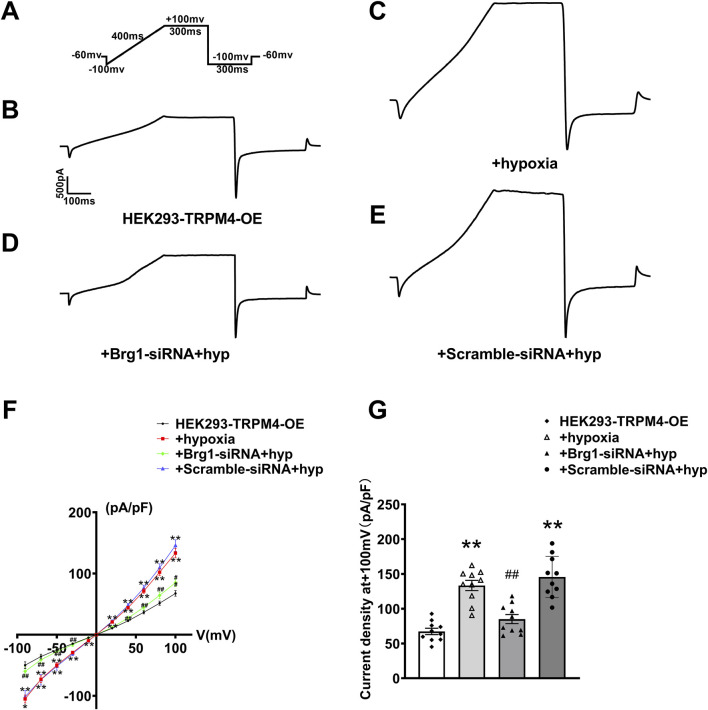
Brg1 knockdown decreased hypoxia-induced abnormal elevation of TRPM4 current density in HEK293 cells. **(A)** Individual currents recorded using a ramp protocol depicted in the inset. **(B–E)** Typical current trace from HEK293-TRPM4-OE group, HEK293-TRPM4-OE + hypoxia group, HEK293-TRPM4-OE-Brg1-siRNA + hypoxia group and HEK293-TRPM4-OE-Scramble-siRNA + hypoxia group. **(F)** Current-voltage (*I-V*) relationship curves of TRPM4 from each group. **(G)** Comparation of TRPM4 current density at +100mv from each group. Statistical analysis was performed with one-way ANOVA. ^**^ *p* < 0.01 vs. HEK293-TRPM4-OE group; ^##^ *p* < 0.01 vs. +Scramble-siRNA + hypoxia group. n = 10 in each group.

Next, we used the whole-cell patch-clamp technique to measure hypoxia-induced TRPM4 current in cardiomyocytes. It has been reported that a hypoxia model was established by incubation in a hypoxic incubator for 12 h (Simard et al., 2012; Salazar et al., 2021; So et al., 2016). Displayed within the figures are the stimulation protocol (Figure 7A), alongside current traces for various groups: HEK293 cells overexpressing TRPM4 (HEK293-TRPM4-OE) (Figure 7B), HEK293-TRPM4-OE cells subjected to hypoxia (Figure 7C), HEK293-TRPM4-OE cells treated with Brg1-siRNA under hypoxic conditions (Figure 7D), and HEK293-TRPM4-OE cells treated with scramble-siRNA under hypoxic conditions (Figure 7E). These recordings revealed a significant escalation in TRPM4 currents in response to hypoxia, compared to the baseline established by HEK293 cells expressing TRPM4 alone.

Importantly, it was noticed that the knockdown of Brg1 could significantly reduce the rise in TRPM4 currents induced by hypoxia. This modulation is quantitatively supported by the measured *I-V* curve normalization and current density across the four cell groups at +100mv (Figures 7F, G). The data explicitly showed that hypoxia elevated TRPM4 current density, and the phenomenon that was notably mitigated by Brg1 suppression. These findings illuminated Brg1’s crucial role in modulating both the expression and function of TRPM4 under hypoxic.

Collectively, the above data demonstrated that Brg1 inhibition moderated the abnormal elevation of TRPM4 induced by hypoxia. This understanding not only broadened our knowledge of Brg1’s regulatory mechanisms under stressful circumstances but also underlined its therapeutic significance in lessening cardiac dysfunctions related to hypoxia.

### 2.8 Brg1 modulates TRPM4 promoter activity via interaction with RUNX1

**FIGURE 8:**
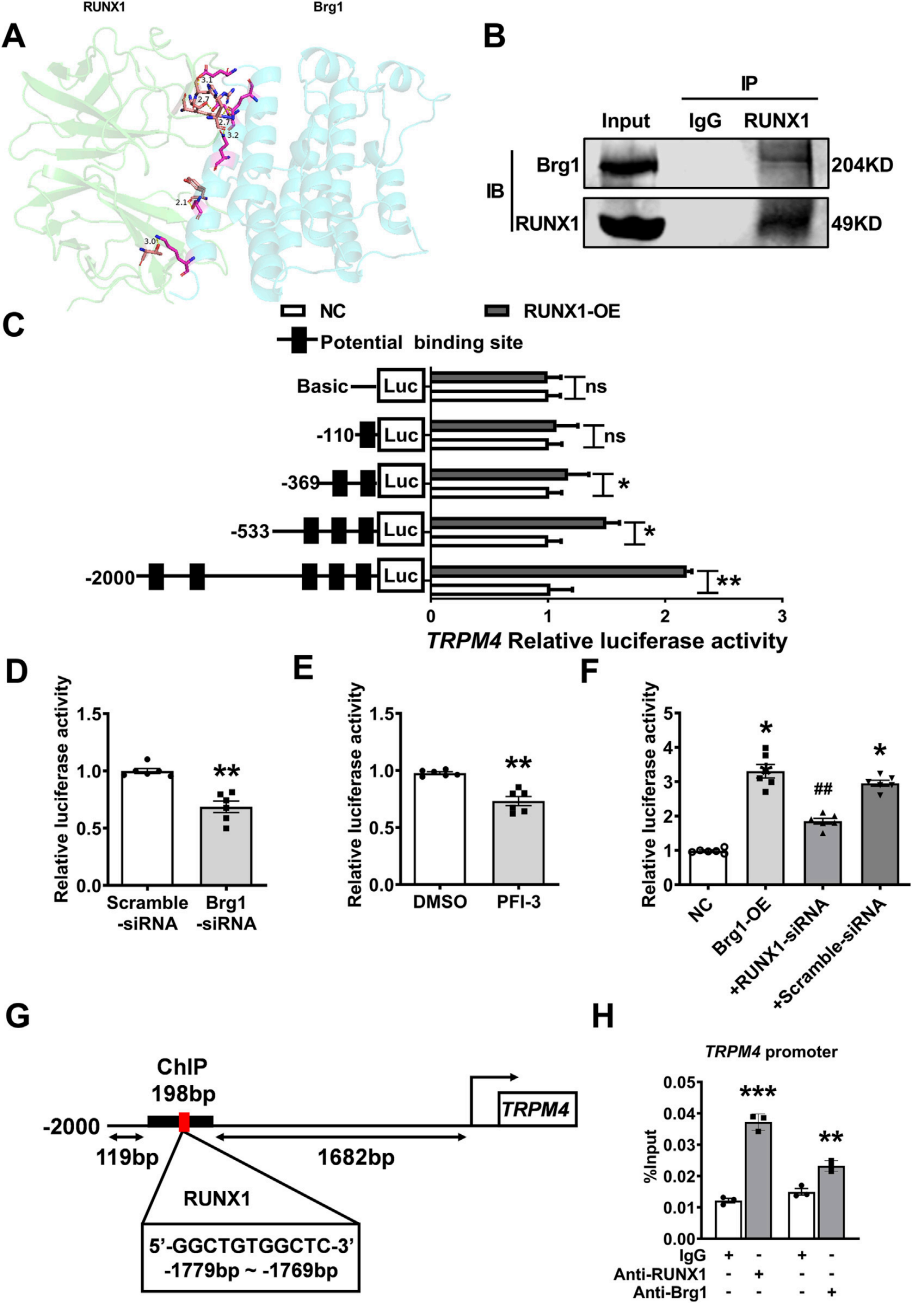
Brg1 modulated TRPM4 promoter activity by interaction with RUNX1. **(A)** Predicting the residue label and hydrogen bond length of interaction between Brg1 and RUNX1, and the combination state and surface structure of Brg1 and RUNX1 by Protein Data Bank. **(B)** Co-immunoprecipitation assays of Brg1 and RUNX1 on the nucleoprotein extracted from neonatal mouse cardiomyocytes. **(C)** RUNX1 significantly boosted TRPM4 promoter activity. Statistical analysis was performed with two-tailed Student’s t-test. *p < 0.05, ^**^ *p* < 0.01 vs. NC group. n = 8 in each group. **(D)** Brg1 knockdown significantly decreased the TRPM4 promoter activity. ^**^ *p* < 0.01 vs. Scramble-siRNA group by two-tailed Student’s t-test. n = 6 in each group. **(E)** Inhibited Brg1 by PFI-3 (10 μM) significantly reduced the TRPM4 promoter activity. ^**^ *p* < 0.01 vs. DMSO group by two-tailed Student’s t-test. n = 6 in each group. **(F)** RUNX1 knockdown declined the increases in TRPM4 promoter activity caused by Brg1-OE. ^*^ *p* < 0.05, vs. NC group; ^##^ *p* < 0.01 vs. +Scramble-siRNA by one-way ANOVA analysis. n = 6 in each group. **(G)** A ChIP assay showed the binding site of RUNX1 an s c2d TRPM4 promoter. **(H)** qRT-PCR of the ChIP products demonstrated that both Brg1 and RUNX1 were bound to the TRPM4 promoter region, IgG served as an antibody control. ^**^ *p* < 0.01 and ^***^ *p* < 0.001 vs. IgG group by a two-tailed Student’s t-test. n = 3 in each group.

In fact, Brg1 functioning as a molecular chaperone, it is necessary that interacted with transcription factors to regulate the transcription of downstream target genes—a process integral to gene transcription, replication, and recombination (Ge et al., 2017; Matsumoto et al., 2016). To decode the mechanism of Brg1’s influence on TRPM4, database prediction tools such as STRING and Gene MANIA were utilized to identify transcription factors that interact with Brg1. The Protein Data Bank (PDB) further aided in delineating the interaction site and structure between Brg1 and RUNX1 (Figure 8A). It has been reported that expression of RUNX1 has been increased in myocardial hypoxia and myocardial infarction, accompanied interactions and co-localization with Brg1 (Bakshi et al., 2010; Riddell et al., 2020; McCarroll et al., 2018). The interaction between Brg1 and RUNX1 was substantiated through Co-immunoprecipitation testing (Figure 8B).

The binding status of RUNX1 within the TRPM4 promoter region (2000bp upstream from TRPM4 transcription initiation site) was assessed using the JASPAR database, and we found multiple high-scoring potential binding sites within the TRPM4 promoter region (Figure 8C). Then we transfected reporter genes containing the TRPM4 promoters into HEK293 cells to detect the regulatory effect of RUNX1 and Brg1on TRPM4 promoter activity by dual luciferase reporter assays. The results elucidated RUNX1 augmented TRPM4 promoter activity (Figure 8C). While Brg1 knockdown via small interfering RNA and the Brg1 inhibitor PFI-3 significantly reduced TRPM4 promoter activity in comparison to control (Figures 8D, E). Conversely, Brg1 overexpression markedly boosted TRPM4 promoter activity, a process partially reversed by RUNX1 knockdown (Figure 8F). Chromatin immunoprecipitation (ChIP) assays further highlighted RUNX1 and Brg1 clustered on TRPM4 promoter region (−1881 to −1683bp from TRPM4 transcription initiation site) (Figures 8G, H).

**FIGURE 9:**
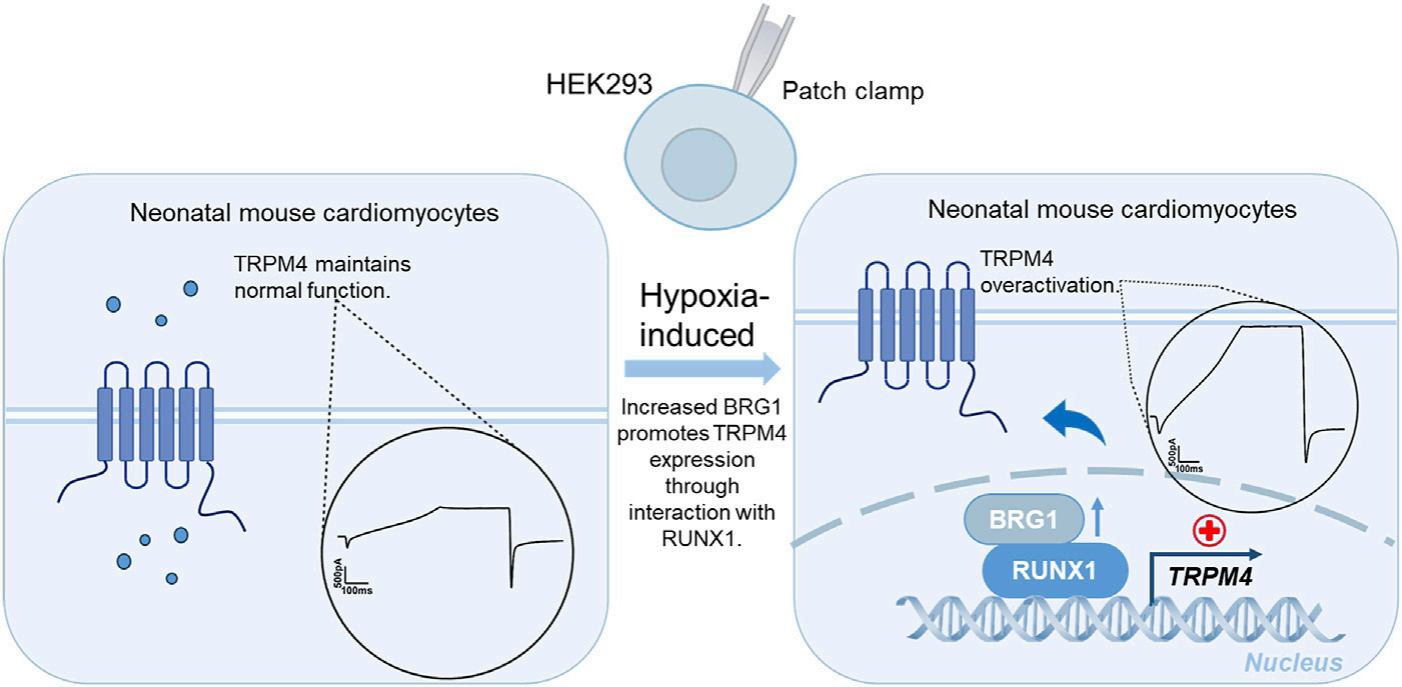
A schematic diagram summarizing the potential mechanisms that Brg1 interacted with RUNX1 transcriptional regulated TRPM4, that eventually leads to TRPM4 overactivation in cardiomyocytes induced by hypoxia.

Cumulatively, these results illuminated that Brg1’s regulation of TRPM4 promoter activity through RUNX1 interaction was a critical mechanism for gene transcription in the cardiac context (Figure 9).

## 3 Discussion

The Transient Receptor Potential Melastatin 4 (TRPM4) channel is extensively distributed within cardiac tissue, playing an indispensable role in myocardial cell development (Guinamard et al., 2004; Simard et al., 2020). And the dysregulation of TRPM4 is implicated in various cardiac conduction system disorders (Castaldo et al., 2017; Runte et al., 2017; Stallmeyer et al., 2012; Amarouch and El Hilaly, 2020). Evidence from animal models illustrates that TRPM4 expression escalates under conditions such as myocardial ischemia-reperfusion, myocardial infarction, and hypoxia-reoxygenation. Notably, inhibition of TRPM4 has shown promise in mitigating arrhythmias induced by hypoxia-reoxygenation in mice and in offering protection against ischemia-reperfusion injury in rat hearts, underscoring its therapeutic potential in cardiovascular disease management (Simard et al., 2012; Pironet et al., 2019). However, there are fewer reports about mechanism of TRPM4 regulation. In this study, we elucidated for the first time a novel regulatory mechanism of Brg1 and RUNX1 synergistically modulating TRPM4 activity.

Brg1, functioning as an essential constituent of the SWI/SNF chromatin remodeling complex, assumes a crucial role in the regulation of gene transcription. It achieves this by modifying the chromatin architecture, thereby exerting an influence on the transcriptional initiation and elongation procedures (Barker et al., 2001; Debril et al., 2004; Harada et al., 2015). In cardiac stress conditions, Brg1 expression is known to increase, contributing to myocardial hypertrophy induced by pressure overload. Genetic interventions targeting Brg1 have yielded beneficial outcomes, including the attenuation of myocardial hypertrophy, reduced neutrophil recruitment, and improved myocardial ischemia-reperfusion outcomes in animal models (Hang et al., 2010; Shao et al., 2020). Our previous study found that Brg1 expression was increased in myocardial infarction and Brg1 regulated the transcriptional levels of sodium and potassium channels, thereby reducing the incidence of arrhythmia post-infarction (Li et al., 2024). These evidences showed Brg1 not only as a pivotal regulator of cardiac gene transcription but also as a potential therapeutic target in cardiovascular diseases. The association between Brg1 and TRPM4, especially in hypoxic conditions, suggested a complex regulatory network that could lead to new interventions for dysregulated TRPM4 expression. The elucidation of this relationship offers insightful perspectives into the molecular mechanisms underlying cardiac pathologies and introduces promising avenues for therapeutic exploration.

Previous studies (Liu et al., 2010; Hof et al., 2017; Liu et al., 2013), have extensively reported that abnormal TRPM4 expression is associated with myocardial depolarization and arrhythmias. Our previous study demonstrated that Brg1 expression was increased in myocardial infarction and prolongs cardiac conduction and action potential duration, which correlate with alterations in ion channels, depolarization, and gap linkage. Considering the abnormal expressions of Brg1 and TRPM4 in myocardial hypoxia conditions. This prompted us to investigate their potential relationship and subsequent effects on cardiac function. We found that knockdown or overexpression of Brg1 positively regulated TRPM4. It’s known to all, Brg1 often inhibited or activated downstream target gene with transcription factors in cardiovascular disease. Subsequently, database analyses, such as those conducted using STRING, Gene MANIA, and PDB, revealed the potential interaction between Brg1 and the transcription factor RUNX1. Immunoprecipitation experiments validated this interaction. Evidence indicated an increase of the transcription factor RUNX1 under pathological conditions such as myocardial infarction and dilated cardiomyopathy, suggesting its pivotal regulatory role (Li et al., 2019; Ni et al., 2021).

Subsequently, we predicted multiple binding sites for RUNX1 with the promoter region of TRPM4, located in 2000bp upstream of TRPM4 transcription initiation site utilizing JASPAR databases. The results of dual luciferase reporter gene assays demonstrated Brg1 could significantly alter the activity of TRPM4 promoter, the process was partially reversed by RUNX1 knockdown. Moreover, Brg1 was also detected in the RUNX1-enriched promoter region of TRPM4 by ChIP assay. Above results pointed towards a regulatory mechanism that Brg1 influences the TRPM4 channel activity through interaction with RUNX1.

Furthermore considering the diversity of ion channels in cardiomyocytes and the fact that TRPM4 channels are permeable to a wide range of ions, in order to exclude this interference, a HEK293 cell line expressing TRPM4 (referred to as TRPM4-HEK293 cell) was developed to isolate and directly measure the current of the TRPM4 channel. This model minimized the impact of other ions such as Na^+^, K^+^, and Ca^2+^, ensuring the recorded currents accurately reflected the functionality of the TRPM4 channel (Earley and Brayden, 2015). To investigate whether Brg1 regulates TRPM4 transcriptional levels, we designed a plasmid containing the full-length sequence of the TRPM4 promoter region for transfection into HEK293 cells. The investigation demonstrated that modulation of Brg1, either through overexpression or knockdown, affected the current of the TRPM4 channel, emphasizing the role of Brg1 in regulating the expression and functionality of TRPM4 channels in cardiomyocytes.

Our findings are the first to show Brg1’s regulatory role in TRPM4 transcription, introducing a new dimension to its function in myocardial cells. It delves into the potential mechanism through which Brg1 exerts transcriptional control over the TRPM4 channel by binding with the transcription factor RUNX1, providing foundational insights for future research. However, further validation in disease model were still required. These findings not only broaden our comprehension of the TRPM4 channel but also open up new pathways for developing treatments targeting myocardial hypoxia. Consequently, this research has the potential to usher in new therapeutic strategies in the realm of clinical investigations, thereby highlighting the crucial importance of the interaction between Brg1 and RUNX1 within the mechanisms of cardiovascular diseases.

## 4 Conclusion

This study highlighted Brg1’s critical regulatory role in TRPM4 transcription in myocardial cells, showing that changes in Brg1 expression directly affect TRPM4 function. Our findings showed that Brg1 modulates TRPM4 channel activity, especially under hypoxic conditions relevant to heart diseases. Through overexpression and knockdown studies, we found that Brg1 markedly influenced TRPM4 expression, channel currents, and membrane potentials, emphasizing its importance in cardiac electrophysiology. Additionally, the newly identified Brg1-RUNX1 interaction illustrated the complexity of TRPM4 regulation in cardiac disease. This research identified the Brg1-TRPM4 axis as a promising therapeutic target for myocardial hypoxia-related cardiovascular diseases, providing new directions for clinical studies.

## 5 Materials and methods

### 5.1 Culture of neonatal mouse cardiomyocytes and treatments

Primary cultures of neonatal mouse myocytes, from 1 to 2 day-old mouse. Immediately after euthanasia of mouse pups, hearts were removed, and myocytes were isolated with 1.0 mg/mL collagenase type II (ThermoFisher Scientific). Isolated myocytes were collected at 10 min intervals until complete tissue digestion. The cells were resuspended in Dulbecco’s modified Eagle’s medium (high glucose) containing 10% fetal bovine serum (FBS) and 1% penicillin-streptomycin solution, and plated in culture dishes for 90 min to allow attachment of fast-adherent fibroblasts. Myocytes were collected and plated in a 6-well cell culture plate at 37°C environment with 5% CO_2_. HEK293 cells were also cultured in the same way. To induce hypoxia, the cells were cultured in anoxic incubator for 12 h. To inhibit Brg1, the cells were treated with 10 μM PFI-3 (Sigma-Aldrich, Saint Louis, United States), a Brg1 inhibitor, for 24 h.

### 5.2 Cell transfection

Cells with a density of 70%–80% were transfected. After 24 h of culture in the plates, the culture medium is changed to serum-free medium for transfection. The Lipo 2000 liposome transfection kit (Lipofectamine 2000 Transfection Reagent, Thermo Scientific, Boston, United States) was used for transfection of Brg1, TRPM4 overexpression plasmids and siRNA-Brg1 or negative control siRNA. After 6 h, the culture medium was changed, and the culture continued for 24 h for the follow-up experiments.

### 5.3 Western blot

Total proteins were extracted from cultured cells with the RIPA Lysis Buffer (Beyotime, China). The proteins were resolved on 8% SDS-PAGE gels (Beyotime Biotech), then transferred onto nitrocellulose membranes (PALL Gelman). After incubation in 5% non-fat milk for 1.5 h at room temperature, the membranes were incubated at 4°C overnight with TRPM4 antibody (1:500; ABclonal Technology Co.,Ltd.), β-actin antibody (1:1000; Santa, United States), Brg1 antibody (Sigma-Aldrich, 07–478, 1:500) and RUNX1 antibody (Proteintech 25315-1-AP, 1:1000). The membrane was washed with PBS-T (Phosphate Buffered Saline with Tween-20), and incubated at room temperature with secondary antibody (1:10000) for 55 min. Blots were detected with the Odyssey infra-red imaging system (LI-COR, United States, #ODY-3149). Band intensities were quantified using Odyssey Version 3.0. X software for each group and normalized to Actin band intensity.

### 5.4 Co-immunoprecipitation assay

After protein extraction, the protein concentration should be determined. Each IP sample were added to 200–500 μg of cell lysate with a concentration of approximately 1 μg/μL. First, proteins and antibody were incubated overnight, and then appropriate antibody were selected for IP. Refer to the antibody instructions for dosage information. Add 500 μg (the amount of cell lysate depends on the situation) of cell lysate to a 1.5 mL EP tube, add the IP antibody, then add IgG to 500 μg of cell lysate and rotate and mix at 4°C overnight. Add 20 μL of Protein A or Protein G bead slurry to 200 μL of cell lysate, and rotate and mix at 4°C overnight. Binding/washing buffer was used to resuspend the beads sufficiently, the beads was separated magnetically and the supernatant was discarded. Repeat washing three times. Adding SDS-PAGE Loading Buffer into the EP tube, mix well, and heat at 95°C for 10 min. Separate the magnetic beads, collect the supernatant, and perform SDS-PAGE detection.

### 5.5 qRT-PCR

Total RNA of cells was extracted using Trizol reagent (Invitrogen, United States), synthesis of complementary DNA (cDNA) was obtained using high-capacity cDNA reverse transcription kit (Toyobo, Japan). Real-time quantitative PCR (qRT-PCR) was performed using SYBR Green Master (Rox) (Sigma-Aldrich) on the ABI7500 Fast Real-Time PCR system (Applied Biosystems, United States). The primer sequences used to amplify the cDNA of these genes are as follows, the glyceraldehyde 3-phosphate dehydrogenase (GAPDH) RNA was amplified as an internal control. The relative gene expression level (the amount of tar-get, normalized to the endogenous control gene) was calculated using the comparative Ct method formula: 2^−ΔΔCT^. Brg1 (forward): 5′-GGT​TCT​GCC​CAC​AGC​ATG​AT-3'; Brg1 (reverse): 5′-GGA​CTC​CAT​AGG​CTT​GTG​CAT-3'. TRPM4 (forward): 5′-GGA​CTG​CAC​ACA​GGC​ATT​G-3'; TRPM4 (reverse): 5′-GTA​CCT​TGC​GGG​GAA​TGA​GC-3'. GAPDH (forward): 5′-AAG​AAG​GTG​GTG​AAG​CAG​GC-3'; GAPDH (reverse): 5′-TCC​ACC​ACC​CAG​TTG​CTG​TA-3'.

### 5.6 Immunofluorescence staining

Myocytes cells were dispersed on a 14 mm glass slide in the center of the culture plate, PBS was used to wash these cells for three times. Cells were fixed with 4% paraformaldehyde for 15 min, penetrated with 0.4% Triton for 55 min and blocked with 10% goat serum at room temperature in the dark for 55 min. Cells were then incubated with TRPM4 antibody (1:250; ABclonal Technology Co. Ltd.) and α-actinin antibody at 4°C overnight. After washing with PBS, incubated with Alexa Fluor 488, Alexa Fluor 594 secondary antibody for 55 min at room temperature (1:300; Proteintech, United States) and incubated with DAPI (Biosharp) for 15 min. Fluorescence was visualized with an inverted Zeiss LSM 800 (Germany) fluorescence confocal microscope. All imaging was processed in an identical manner to capture the real time images of each sample, images obtained by ZEN and ImageJ analysis.

### 5.7 Membrane potential detection

Potentiometric fluorescence dye DiBAC4(3) (AAT Bioquest, United States) was measured to membrane potential., primary cultured cells were loaded with 100 nmol/L DiBAC4(3) at 37°C for 30 min (Kwan et al., 2009). Then cells were washed with serum-free culture medium for 3 times and the fluorescence was measured by using Zeiss LSM 800 fluorescence confocal microscope. The membrane potential was represented by the relative intensity of fluorescence.

### 5.8 Solution

For patch-clamp experiments in whole-cell configuration, Using MF-830 microelectrode polishing machine (Japan), the borosilicate glass capillaries (Sutter instrument, United States) drawn by P-97 microelectrode pulling instrument (Sutter instrument, United States) were polished. The glass pipettes (tip resistance, 2–3 MΩ) were filled with an intracellular solution containing (in mM): 156 CsCl, 8 NaCl, 1 MgCl_2_⋅6H_2_O, 10 HEPES (pH 7.2 with CsOH), and the free Ca^2+^ concentration at 100 μM with CaCl_2_ using WEBMAXCLITE program (http://www.stanford.edu/∼cpatton/downloads.htm). Extracellular solution contained (in mM): 156 NaCl, 1.5 CaCl_2_, 10 Glu⋅H_2_O, 10 HEPES, (pH 7.4 with NaOH) (Amarouch et al., 2013; Syam et al., 2014).

### 5.9 Patch clamp electrophysiology

Membrane currents were recorded with a Multi-Clamp 700B amplifier (Axon Molecular Devices, United States) controlled by Clampex 10.6 via a Digidata 1440A (Axon Molecular Devices, United States). Data were low-pass filtered at 1 kHz and sampled at 5 kHz. Patch-clamp recordings were carried out in the whole-cell configuration at room temperature. TRPM4 currents were investigated using a ramp protocol. The holding potential was −60 mV. The 400 ms increasing ramp from −100 to +100 mV ended with a 300 ms step at +100 mV and then 300 ms at −100 mV. A new ramp was performed every 2 s (Syam et al., 2016). Currents were analyzed with Clampfit 10.6 software (Axon Molecular Devices, United States). Current densities were obtained by dividing the peak current recorded at 100 mV by the cell capacitance.

### 5.10 Dual-luciferase reporter gene assay

HEK293 cells were cultured and then transfected with a luciferase chimeric vector containing a promoter 2000 bp upstream of TRPM4, along with Brg1-siRNA and control Scramble-siRNA. Additionally, cells were treated with 10 μmol/L PFI-3, Brg1 overexpressed plasmid, RUNX1-siRNA, and Scramble-siRNA as a control. The fluorescence intensity of luciferase catalyzed by the transfected chimeric vector was measured using a GLOMAX 20/20 fluorescence detector to study the effect of RUNX1 on transcription activity of the TRPM4 promoter.

### 5.11 Chromatin immunoprecipitation (ChIP) assay

ChIP assays were performed by ChIP assay kit materials (Thermo Scientific, MA, United States). The neonatal mouse ventricular cardiomyocytes were subjected to the ChIP assays using anti-Brg1 (Proteintech, IL, United States), anti-RUNX1 (Proteintech) antibodies, or rabbit IgG (Cell Signaling Technology, Danvers, MA, United States). DNA was immunoprecipitated from the sonicated cell lysates using Brg1, RUNX1, or IgG antibody and subjected to PCR to amplify the binding sites.

### 5.12 Statistics

The data were processed with GraphPad Prism 9.4.1 analysis software and group data are presented as mean ± SEM. The statistical significance of differences was assessed by using ANOVA one way analysis or a two-tailed Student *t*-test and *p* < 0.05 was taken to indicate a statistically significant difference.

## Data Availability

Publicly available datasets were analyzed and/or generated in this study. These can be accessed at: https://doi.org/10.6084/m9.figshare.27890805.v1; https://doi.org/10.6084/m9.figshare.27924105.v1; https://doi.org/10.6084/m9.figshare.27890946.v1; https://doi.org/10.6084/m9.figshare.27890976.v1; https://doi.org/10.6084/m9.figshare.27890991.v1; https://doi.org/10.6084/m9.figshare.27891018.v1; https://doi.org/10.6084/m9.figshare.27924147.v1. Further inquiries can be directed to the corresponding author.
