# Supplementary material for: Brg1 and RUNX1 synergy in regulating TRPM4 channel in mouse cardiomyocytes
Source: Front Pharmacol. 2024 Dec 12;15:1494205. doi: 10.3389/fphar.2024.1494205 (PMC11669506; doi:10.3389/fphar.2024.1494205)

Fig 1A protein bands, red areas are images presented in the text

Maker, Ctl, Brg1-OE, Ctl, Brg1-OE, Maker, Ctl, Brg1-OE, maker

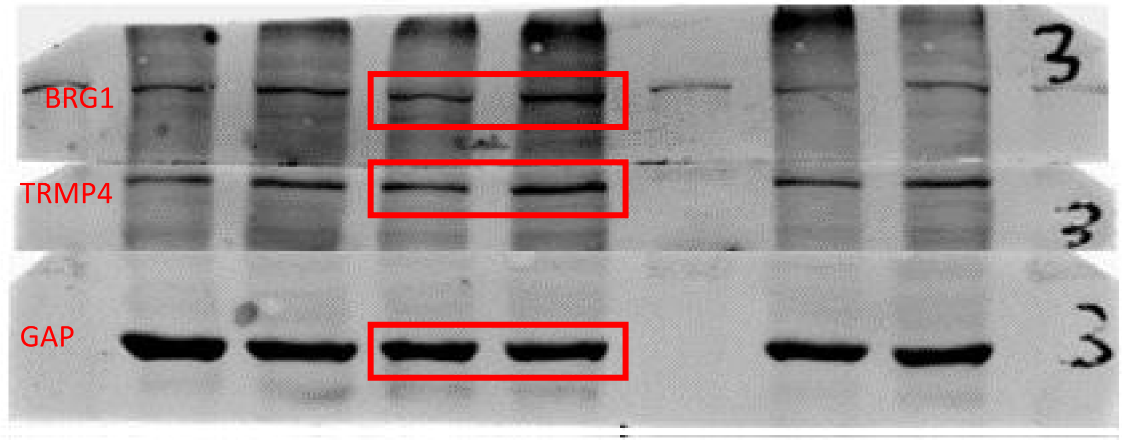

Fig 2A protein bands, red areas are images presented in the text

Maker, Scramble-siRNA, Brg1-siRNA, maker, Scramble-siRNA, Brg1-siRNA, Scramble-siRNA, Brg1-siRNA, maker

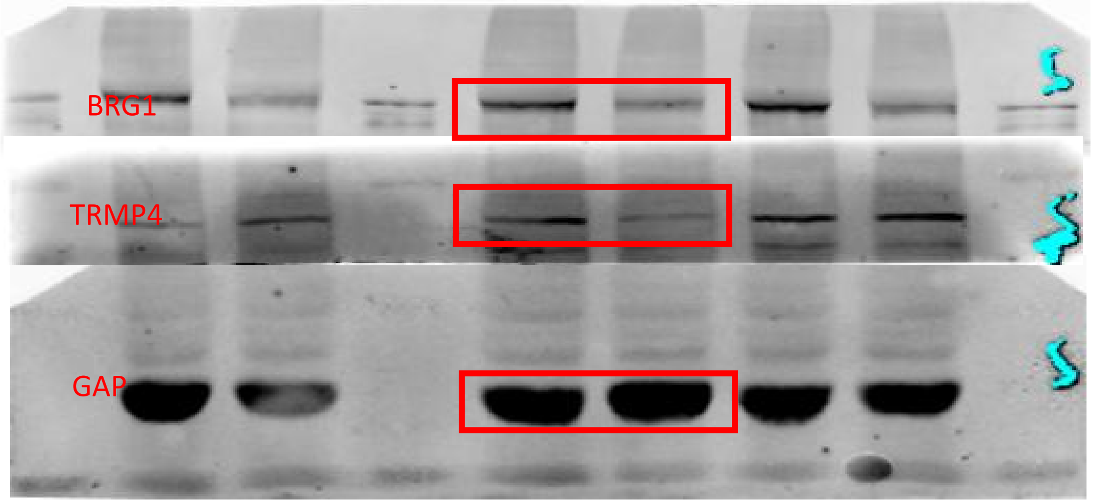

Fig 2D protein bands, red areas are images presented in the text

Maker, DMSO, PFI-3, Maker, DMSO, PFI-3, DMSO, PFI-3, maker

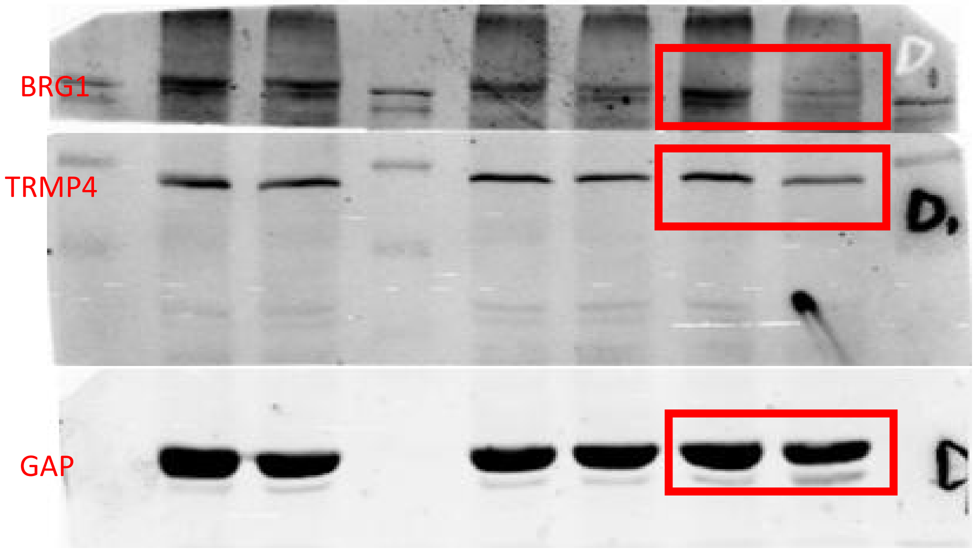

Fig 4A protein bands, red areas are images presented in the text

TRMP4-OE - + - +

Maker maker

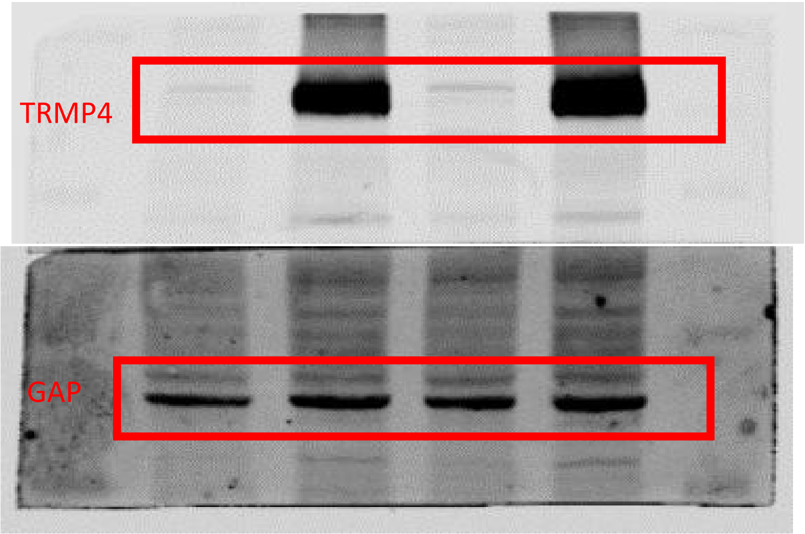

Fig 6A protein bands, red areas are images presented in the text

Maker, ctl, hyp, +Brg1-siRNA, +Scramble-siRNA, ctl, hyp, +Brg1-siRNA, +Scramble-siRNA, maker

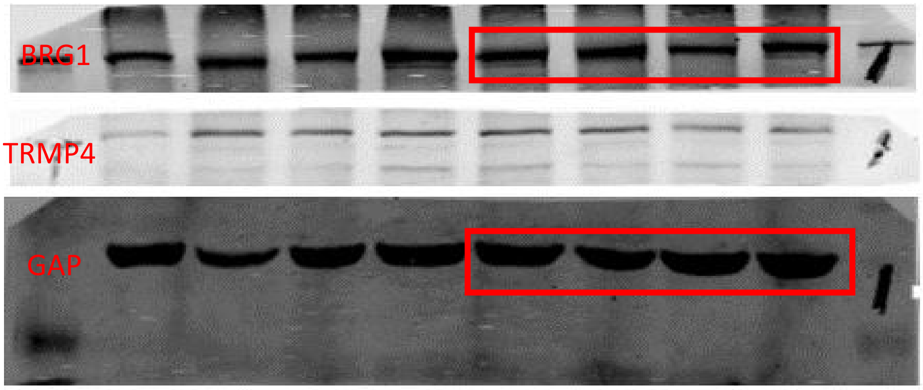

Maker, ctl, hyp, +Brg1-siRNA, +Scramble-siRNA, ctl, hyp, +Brg1-siRNA, +Scramble-siRNA, maker

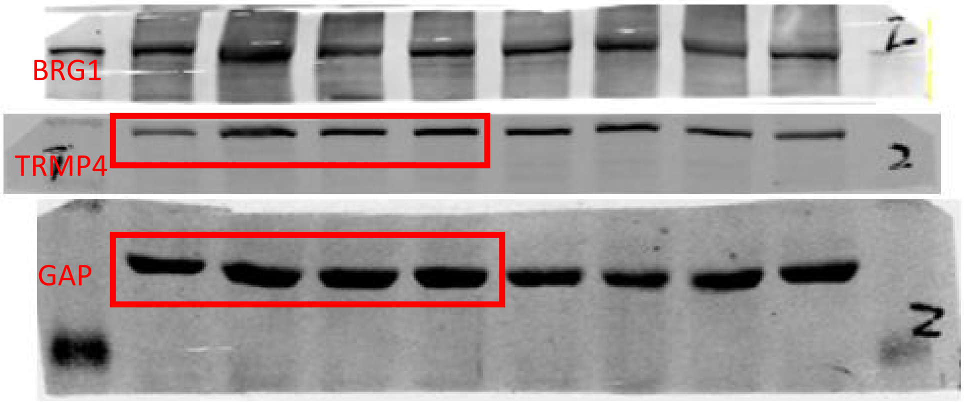

Fig 8B protein bands, red areas are images presented in the text

			IP	IP		IP	IP	
Maker	Maker	input	igG	RUNX1	input	igG	RUNX1	maker

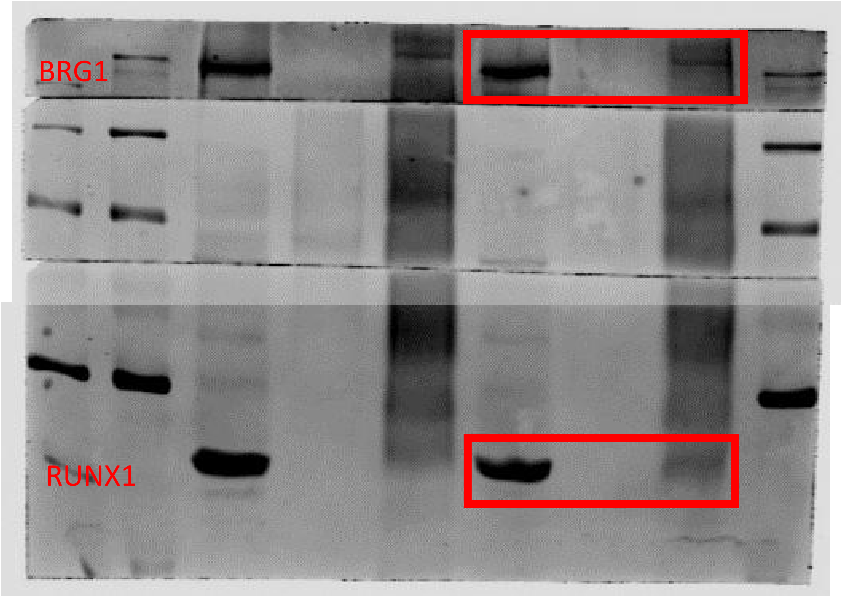

Supplement: Supplementary file 1 [file DataSheet1.pdf]
